# Mothers Make a Difference: Mothers Develop Weaker Bonds with Immature Sons than Daughters

**DOI:** 10.1371/journal.pone.0154845

**Published:** 2016-05-18

**Authors:** Lars Kulik, Doreen Langos, Anja Widdig

**Affiliations:** 1 Junior Research Group of Primate Kin Selection, Department of Primatology, Max-Planck Institute for Evolutionary Anthropology, Deutscher Platz 6, 04103 Leipzig, Germany; 2 Institute of Biology, Faculty of Bioscience, Pharmacy and Psychology, University of Leipzig, Talstrasse 33, 04103 Leipzig, Germany; Eunice Kennedy Shriver National Institute of Child Health and Human Development, UNITED STATES

## Abstract

Among mammals, individuals form strong social bonds preferentially with their kin. Differences in these relationships are linked to differential kin availability due to sex-specific dispersal patterns, but there is some indication that differential bonding among sexes already occurs prior to maturation. However, little is known about how these patterns arise during individual development. Here we investigated sex differences in the development of mother-offspring bonds in rhesus macaques (*Macaca mulatta*). Our results revealed that mothers showed sex-biased bonding toward their offspring. Sons had a distinctly higher probability of receiving aggression from their mothers than did daughters in the first year of life, while no differences were found with respect to affiliative interactions. After the first year, probabilities of all affiliative and aggressive behaviours investigated were higher for daughters than for sons, although generally declining. Furthermore, sons spending less time with their mother and receiving more maternal aggression tended to disperse earlier. The results of our study suggest that mothers influence their bonding strength with offspring by interacting less affiliative with sons than daughters.

## Introduction

Many mammal species live in social groups that are characterized by differentiated social relationships among individual group members [1]. Preferential relationships, often called bonds, have been shown to have an enormous impact upon individual fitness [2]. While several studies have reported that such preferential bonding also occurs among nonrelatives [3], there is strong evidence for bonds being established mainly among close relatives [4].

In particular, maternal relatedness among the philopatric sex seems to be a driver of strong and long-lasting social bonds [4–6]. These findings led to the assumption that the philopatric sex should form more and stronger social bonds than the dispersing sex [7–9]. Indeed there is evidence that the members of the dispersing sex show less social bonding after they have left their natal group (e.g. males [10], females [11]). However, it has been reported that even in the absence of potential kin partners, the dispersing sex is able to form strong social bonds in their new social group, at least in some species [12,13].

Although the theoretical and empirical evidence for the establishment of social bonds implicitly focuses on the period after migration, there is some indication that sex differences in affiliative patterns can already be observed prior to sexual maturity [14]. Particularly in female-philopatric primates, juvenile females were engaged in more social interactions than males in general, with females tending to interact more with females and males more with males (e.g. [15,16]). Similar patterns have been found in other female-philopatric mammals. For example, among African elephants, juvenile females affiliated more with their family while juvenile males preferred non-family members [17]. Likewise, juvenile female spotted hyenas tended to be spatially closer to their family than were males [18]. In contrast, in male-philopatric chimpanzees, juvenile males interacted with more partners than did juvenile females [19] and adolescent males were found to be more gregarious than adolescent females [20]. Similarly, adolescent males in male-philopatric spider monkeys were generally more affiliative and social than females [21].

Moreover, there is evidence that mother-offspring bonds show a sex-dependent bias in female-philopatric mammals, such that juvenile females show a more intense bond with their mother than juvenile males (e.g. [22,23]). In male-philopatric muriquis, however, it was shown that juveniles of both sexes maintained equally close proximity to their mother [24]. In chimpanzees, no sex difference was found in grooming relations between mothers and their juvenile offspring, but sons began to travel independently earlier than daughters (e.g. [20]).

While sex differences in juvenile affiliation patterns have been linked to adult social roles and species-typical social systems, it has been argued that the quality of mother-offspring bonds and early social experiences may also have an impact upon offspring socialization [25–27]. It has been shown that mothering style, described by the degree of protectiveness and rejection [28], has effects on the behaviour of infants. For example, in Japanese macaques the infants of mothers receiving more maternal rejection tended to become independent at an earlier age [29]. In contrast, infants of more protective mothers exhibit delayed independence and more fearful behaviour in novel situations among vervet monkeys [30]. In particular, it was shown in macaques that the mother-infant relationship in the first year of life can have dramatic short- and long-term behavioural consequences [31]. However, how the early social environment, especially interactions with the mother, influences the process of socialization is not well understood [14].

In primates conflicting evidence has been found with regard to sex-specific maternal behaviour [14,25]. Studies focusing on short time periods (the first few months of offspring life) either found no sex-dependent differences in maternal behaviours (e.g. [32,33]), or reported higher rejection rates of sons [34] and higher grooming [35] and proximity rates [36] with daughters, or *vice versa* [37]. The few studies focusing on longer time periods (>1 year) found more consistent patterns, i.e. differences in maternal behaviour depended on offspring sex and these differences increased with offspring age. For example, in female-philopatric rhesus macaques, mothers displayed higher grooming rates toward daughters than toward sons from three years onwards [23]. Similarly, Japanese macaque mothers showed higher affiliation rates toward daughters than toward sons, beginning at four years of age [38]. However, to date no study addressed differences in maternal behaviour in regard to age and sex of offspring including both aggressive and affiliative interactions covering the entire developmental period.

Here, we studied the development of mother-offspring bonds from birth to maturation (0–4 years) to investigate whether maternal behaviour might facilitate different developmental trajectories in relation to offspring sex. We investigate aggressive and affiliative interactions as both might have different impact on the social bond between mothers and their offspring. Furthermore, we investigated whether the mother-son relationship has an impact upon the timing of male natal dispersal. We addressed these questions in a non-human primate species, the rhesus macaque. This is a good model species given that rhesus macaques live in multi-male, multi-female groups and mate with multiple partners [39] on a seasonal basis [40]. They show sex-biased dispersal with males leaving the natal group [41], while females stay and form strong social bonds with their close maternal kin [42]. Mothers contribute the main component of parental care, although fathers do direct some affiliation preferentially toward their own offspring [43]. In contrast to previous studies, we continuously recorded mother-offspring interactions including grooming, aggression, shared proximity and nursing from birth through to sexual maturation. Since rhesus mothers form stronger bonds with their philopatric offspring [23,42], we expected mothers to already show a sex bias toward their daughters during early infancy. Specifically, we expected daughters to receive more maternal affiliation and less aggression than sons.

## Material and Methods

### Study species and population

The study was performed on the rhesus macaque population of Cayo Santiago (CS, 18°9′N, 65°44′W), a 15.2 ha island approximately 1 km from Puerto Rico (USA), which is managed by the Caribbean Primate Research Center (CPRC). Today’s individuals are direct descendants of 409 founder animals captured in India in 1938 [44]. Although no individuals have since been added to the population except through births, genetic analyses from pedigree data have revealed no evidence of inbreeding (Widdig et al. in review). Demographic information such as date of birth and death, sex, group membership and number of maternal kin is provided by the CPRC, and has been continuously recorded since 1956. Females give birth on average to one offspring a year [44], with infants born within the same seasonal cohort differing in age by up to six months. Males disperse from their natal group between three and six years of age [45], predominantly during the mating season [39]. At the time of data collection, six social groups were present on the island. Group sizes ranged from approximately 80 to 300 individuals in total, which is similar to figures reported from wild populations [46].

### Behavioural data

We studied one entire birth cohort (2005) of troop R (N = 26 females and 29 males) beginning the observations immediately after birth and continuing until the focal subjects reached four years of age. Thus we covered 4 years i.e. the entire period from birth to maturation, as males are able of reproducing at around 3.5–4 years of age [44] and females go through their first menarche at the age of around 2.5 years in this population [47]. We collected a total of 3543 focal observation hours (mean ± SD: 64.42 ± 37.33 hrs per subject). Focal observations were evenly distributed across the day, and the observation time was weekly balanced per focal subject. Using a 20 min *focal animal* protocol [48], we continuously recorded grooming and aggression events of focal subjects with their mothers, considering physical aggression (push, hit, grab, bite, attack) as well as non-physical aggression (stare, head-bobbing, vocal/open mouth threat, lunge, charge, chase [49]). Additionally, data on shared spatial proximity (within 2 m radius of focal subject) as well as the activity of the focal animal were taken every four minutes using point time samples (PTS). To control for a potential sex-bias in maternal food supply [50], we estimated the nursing activity of subjects (hereafter: nursing) by counting all PTSs within each protocol where the infant was found nursing [48]. Focal observations were evenly distributed over the day, and observation time was balanced weekly among focal subjects. Data were collected by AW, DL and two field assistants. Subsequent assistants were trained for a total of two months and included interobserver-reliability tests [51]. All assistants reached a reliability ranging from 90 to 97% with their trainer and with an always significant Cohen's Kappa ranging between 0.806 and 1 (calculated with R package “irr” [52]). Data were collected using Psion WorkaboutTM handheld computers and were processed with Observer software (version 5.0, Noldus, NL).

### Maternity confirmation

Maternity assigned from field observations was genetically tested and confirmed for all mother-offspring pairs (N = 55) in the study [see 43 for details]. All offspring and their mothers were recognized on an individual basis using individual and artificial marks, and additionally confirmed by maternal physical contact in the first three months of offspring life.

### Establishing dominance hierarchies

During our study period, focal subjects were observed from birth to around the age of their maturation; however they might not have yet reached full maturation by the end of this study. Therefore, their rank (and that of all other non-adult group members) was assigned according to the rank of their mothers (hereafter maternal rank).

Dominance ranks among adult females were first established from the outcome of dyadic conflicts observed in 1997 [53] using the I&SI method [54]. The hierarchy was continuously updated over time using agonistic interactions observed during our entire study period [Widdig, unpublished data]. Interestingly, the order of adult females within the hierarchy was highly stable over time [Widdig, unpublished data]

Within the hierarchy, we assigned focal subjects directly below their mother inversely to their birth order [Datta, 1988] and confirmed these predicted focal ranks by behavioural observations over time. In all cases except two, subjects reached their predicted rank according to their mother’s rank and birth order [Widdig, unpublished data]. We calculated the maternal rank on a daily basis to control for rank changes due to births and deaths. For each day, we standardized the ranks to a range from 0 to 1 (lowest to highest ranking) to make both hierarchies comparable.

### Ethics statement

This research complies with the current laws and ethical standards of the countries in which this study was conducted. The Cayo Santiago macaques population are provisioned once daily at a number of locations with a commercial monkey diet (on average 0.23 kg/monkey/day), but spend around 50% of their feeding time foraging on natural vegetation [55]. Water is available ad libitum at several drinking fountains throughout the island. In an annual two-months trapping period, conducted by the CPRC (also assisted by DL and AW), individuals completing their first year of life were trapped and marked with identification codes. From specific individuals also physiological samples may be collected for research purposes. For blood samples animals were temporarily anesthetized by CPRC veterinarian using intramuscular injections of Hydrochloride Ketamine (10 mg/ kg body weight) and up to two 2 ml blood samples then drawn via femoral venipuncture. All animal handling procedures were approved and performed in accordance to the rules and requirements of the CPRC and by the Institutional Animal Care and Use Committee (IACUC) of the University of Puerto Rico (protocol No. 4060105).

### Data analysis and statistical tests

To examine whether mothers show different relationships with their offspring depending upon offspring sex and age we applied Generalized Linear Mixed Models, fitted with binomial error structure and logit link function [56]. These types of models are suggested for data involving repeated observations of individuals, to avoid false positives and erroneous significances, overly narrow standard errors or confidence intervals and to allow controlling for potential confounding variables [57,58]. The data analysed comprised all shared proximity, grooming, maternal aggression, and nursing events observed between mothers and their offspring. From these observations, we calculated our response variables (probability of shared proximity, grooming, dyadic aggression, and nursing) in several steps. First, we determined all the mother-offspring dyads for which we collected data on a given day. Second, we calculated how often a mother-offspring dyad interacted per day. Due to the fact that not every dyad interacted on every day, our data distribution was highly skewed towards zero. Hence, it was not possible to analyse the observed count data using models with a Poisson error structure. Therefore we transformed each response variable into a binary variable, by setting all values greater than zero to one (i.e. mother-offspring dyads either interacted or not on a given day). Thus it was possible to calculate models with binomial error structure. This transformation step represents a data reduction, but we consider it the optimal statistical approach for our dataset. To our knowledge, there is currently no alternative implementation which is able to model the occurrence of excess zeros in zero-inflated count data as a function of the predictors. Our final dataset comprised a total of 9793 data points per behaviour. As the main predictor, we included the two-way interaction between focal sex and focal age (linear and squared) in the model since the effect of focal age on the occurrence probability of a behaviour was expected to be non-linear and to differ between sexes. To achieve a reliable model we also incorporated focal sex, focal age and focal age squared as main effects. As control variables, we added the number of available maternal kin per day (up to the grandparental generation and extracted from demographic data, mean ± SD: 10.72 ± 6.55), maternal age, maternal rank and a variable indicating whether the mother currently possessed an infant younger than the focal subject. Finally, to achieve more reliable *P*-values we included the identity of the mother-offspring dyad and the observation day as random effects, together with random slopes for focal age and focal age squared within the random term for mother-offspring dyad [57,58]. We also included an offset term to control for differences in observation effort between focal subjects per day. To control for potential proximity effects (i.e. spending more time close together may enhance the probability of interacting) we ran additional models for grooming, aggression and nursing that included as an offset term the number of PTSs per focal and day in which the subject's mother was in proximity.

To investigate whether the potential influence of focal age and sex upon grooming probability was affected by who initiated grooming (mother or offspring), we also ran a model with grooming as the response and the three-way interaction between focal sex, age (linear and squared) and focal role (initiator or receiver of grooming) as the main predictor. All other terms were the same as described above. The way in which we recorded our data did not allow an analysis of whether mothers or focal subjects were responsible for maintaining spatial proximity.

The data used in the binomial models were likely to show temporal autocorrelation, which could lead to violation of the assumption of independent residuals. To explicitly account for temporal autocorrelation, we included an autocorrelation term for the mother-offspring dyad as a fixed effect. The autocorrelation term was calculated in several steps using an R function written by Roger Mundry (for details, see [43]). First, we ran the model as described above to derive the residuals. Then, separately for each data point, the residuals of all other data points from the same dyad were averaged, with the contribution of the residuals being weighted by their time lag to the particular data point. The weights followed a normal distribution with a mean of zero (i.e. maximum weight at a time lag of zero). The standard deviation was determined by maximizing the likelihood of the model including the autocorrelation term. This was done separately for each of the four models.

Finally, to test whether proximity of mother-son dyads or maternal aggression toward sons is associated with the timing of natal dispersal we ran a Generalized Linear Model with Poisson error structure and log link function [59]. The number of months that males remained in their natal group before dispersal was used as the response. As predictors, we used the number of maternal aggression received and the number of shared proximity events with the mother (as proportion of total observation time per male subject) within the first year of life, as this time period seems to be crucial in the development of social behaviour patterns [31]. Additionally, we incorporated maternal rank as a control variable. Note, that the analysis is bases to 13 males.

The models were implemented in R (version 2.15.3 [60]) using the function “lmer” of the R package “lme4” [61]. Using the R function “anova” from the package “stats” we ran likelihood ratio tests (LRT [62]) to determine the overall statistical significance of the full model by comparing its fit with that of the respective null model, comprising only the random effects, the offset and the autocorrelation term [63]. We tested interactions for their statistical significance by running additional LRTs comparing the fit of the full model with that of a reduced model lacking the specific interaction but including all other terms. The maximization of the likelihood to find the best fitting standard deviations of the weighting function for the autocorrelation term was performed using the R function”optimize” from the package “stats”. Prior to running the initial models, we checked the distributions of all predictors and, as a consequence, square-root transformed focal age and number of maternal kin. In addition, we *z*-transformed all covariates (including the autocorrelation term) to a mean of zero and a standard deviation of one. To check the assumptions of the full models we calculated Variance Inflation Factors (VIF [64]) which indicated that collinearity was not an issue (largest VIF = 4.86). VIFs were determined using the function “vif” of the R package “car” [65], applied to a standard linear model lacking random effects. The Poisson model was additionally tested for overdispersion, which was not an issue (dispersion parameter = 1.299, χ^2^ = 12.995, df = 10, p = 0.224). For each model we assessed model stability by comparing the estimates derived from a model based on all data with those obtained from models in which dyads (or data points in the case of the Poisson model) were excluded one at a time. This procedure indicated that no influential cases were present.

## Results

During our study period, we observed 4947 grooming events (mean ± SD = 0.51 ± 0.18 per focal observation) and 221 aggression events (mean ± SD = 0.02 ± 0.015 per focal observation) within mother-offspring dyads. Grooming was primarily initiated by the mother (71.62% of all events), while all aggressive interactions were directed from mothers towards offspring, with 172 events involving physical and 49 non-physical aggression. Given the relatively low number of non-physical events, we analysed both types of aggression together. Sons received maternal aggression predominantly within the first year of life (i.e. 62% of all aggression observed toward sons over the four-year study period occurred within their first year of life), and mainly occurred spontaneously (69%), i.e. without previous interaction, including nursing, (but they could have been in proximity (at maximum of 5m) between mother and son or any potential interaction partner being observed within the 2 min prior to maternal aggression. Furthermore, maternal aggression toward sons was evenly distributed across the first year. In contrast daughters received most maternal aggression within their second year of life (i.e. 42% of all the aggression received toward daughters over the four-year study period), and this occurred mainly in response to daughters trying to interact with newborn infants (40% of all aggression received in the second year). Additionally, of the 114099 PTS events recorded over all focal subjects in total, offspring were observed in close spatial proximity to their mother in 30189 PTSs (26%) and in further 9724 PTSs the offspring were found being nursed (8.5%).

Overall the results of the GLMMs revealed that the set of predictor variables used had a clear influence on the probability of occurrence of each behaviour tested (LRTs, full versus null model: spatial proximity: χ^2^ = 219.03, df = 9, p<0.001, grooming: χ^2^ = 79.89, df = 9, p<0.001, maternal aggression: χ^2^ = 29.95, df = 9, p<0.001, nursing: χ^2^ = 280.47, df = 9, p<0.001). This was also the case for the models controlled for spatial proximity (LRTs: grooming: χ^2^ = 75.32, df = 9, p<0.001, maternal aggression: χ^2^ = 39.54, df = 9, p<0.001, nursing: χ^2^ = 282.73, df = 9, p<0.001).

**Fig 1:**
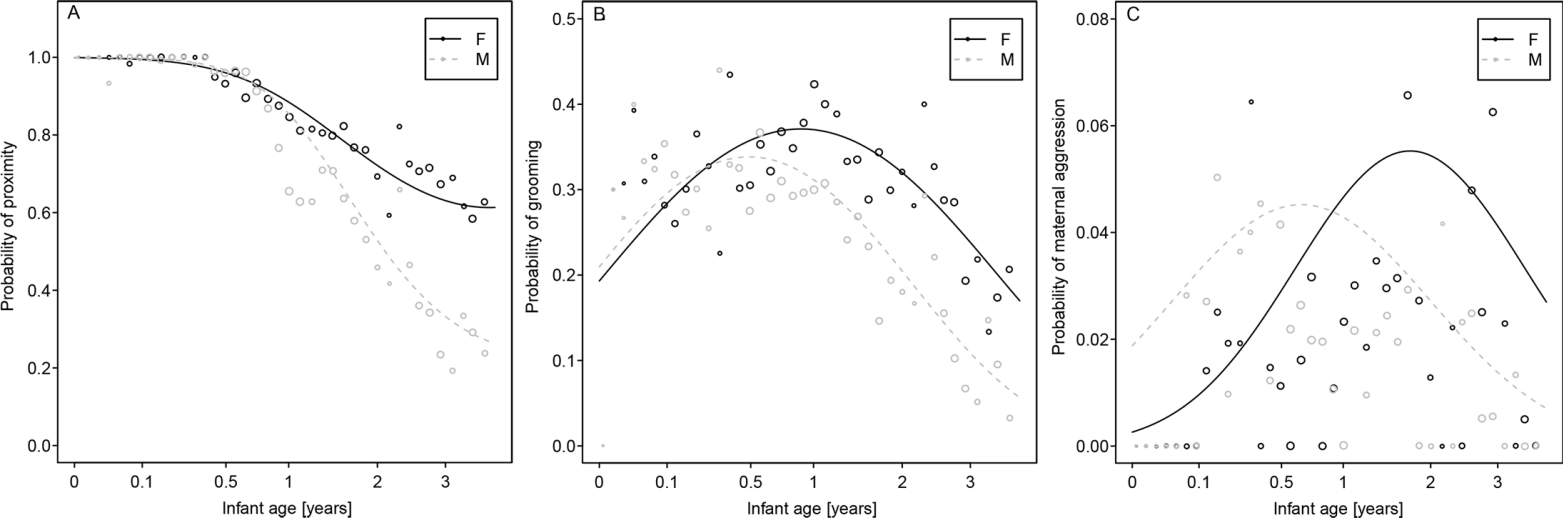
**Effects of focal age and sex on the probability of mother-offspring dyads (A) being in spatial proximity, (B) grooming and (C) maternal aggression toward their offspring.** The lines represent the fitted model. The area of each circle corresponds to the respective number of observations (total N = 9793 per behaviour). The x-axis is square-root transformed.

In more detail, the results showed that the probability of being in spatial proximity to one's mother generally declined over time in both sexes (LRT for the interaction between focal sex and focal age (linear and squared) spatial for proximity: χ2 = 27.94, df = 2, p<0.001). As revealed in Fig 1A, there appeared to be no difference between the sexes with regard to shared proximity with mothers over the first year of life, but commencing around the end of the first year, daughters exhibited a higher probability of being in spatial proximity to their mothers than did sons.

For grooming, the results also revealed a significant interaction between sex and age (LRT for the interaction between focal sex and focal age (linear and squared) spatial for grooming: χ2 = 10.58, df = 2, p = 0.005). While Fig 1B suggests no sex difference within the first few months of life, the probabilities of grooming diverged, reaching a maximum at around 6 months of age for sons and 12 months for daughters. Furthermore, the subsequent decline in grooming probability was more pronounced for sons than for daughters. The model controlled for spatial proximity revealed a similar result (LRTs: grooming: χ2 = 5.05, df = 2, p = 0.080). Furthermore, the relationship between focal age and sex was not influenced by who initiated grooming (three-way interaction: focal sex*focal age (linear and squared)*focal role (initiator or receiver); LRT: χ2 = 0.93, df = 2, p = 0.627).

A completely different pattern emerged for aggressive interaction, which were all initiated by the mother (Fig 1C). The probability of receiving maternal aggression increased over the first year of life in both sexes (LRT for the interaction between focal sex and focal age (linear and squared) spatial for aggression received: χ2 = 12.64, df = 2, p = 0.002), but was initially higher for sons than daughters. Interestingly, this pattern changed after the first year of life, when the probability of receiving maternal aggression decreased noticeably for males. Notably, at the same time, mother-son bonds as measured by means of grooming and shared spatial proximity had already decreased (see above). However, maternal aggression continued to increase for females until the second year of life. In fact, maternal aggression after year one was higher for daughters than sons. The model controlled for spatial proximity revealed a similar result (LRTs: aggression received: χ2 = 9.59, df = 2, p = 0.008).

For nursing we found no sex differences (LRT for the interaction between focal sex and focal age (linear and squared) spatial for nursing: χ2 = 0.090, df = 2, p = 0.956). When controlling for proximity the results remained the same (LRTs: nursing: χ2 = 0.37, df = 2, p = 0.831).

Notably, we found that maternal rank had no influence on the response variable in any of the models (Tables A-E in S1 File).

Furthermore, we found that higher rates of spatial proximity in the first year within mother-son dyads was significantly associated with delayed natal dispersal by sons (LRT: χ^2^ = 15.74, df = 1, p<0.001; Table E in S1 File). Additionally, our data provides some indication that higher rates of maternal aggression received by sons in the first year of life tended to predict shorter tenure in their natal group, i.e. earlier natal dispersal (LRT: χ^2^ = 2.98, df = 1, p = 0.084; Table E in S1 File).

## Discussion

The results of our study show that the development of mother-offspring bonds follows different trajectories for daughters *vs*. sons. Overall, daughters received more maternal affiliation than sons, while our prediction, that daughters should receive less maternal aggression than sons was not fully supported. In more detail, within the first half year of life, shared proximity with the mother, grooming and nursing did not differ with regard to offspring sex, but mothers clearly directed more aggression toward their sons than toward their daughters. After the first year was completed, we found that the probabilities of all behaviours investigated were higher for daughters than sons, although generally declining over time.

The probability of grooming, which was mainly initiated by the mother (71.62%), increased within the first year over time, while spatial proximity between mothers and offspring was naturally high from the beginning. After the first year, grooming and spatial proximity between mothers and offspring generally declined, but more so in males, resulting in higher levels of proximity and grooming between mothers and daughters than between mothers and sons as offspring approached sexual maturity. Interestingly, the grooming probability between mothers and sons began to be lower than that between mothers and daughters at six months of age, which corresponded to the time when sons were most likely to receive maternal aggression. Hence, sons received less grooming and more maternal aggression than daughters at an age that is considered most critical for social development [31]. Daughters, on the other hand, were most often the targets of maternal aggression in their second year of life, when the mother's next infant was already present.

In general, aggression is categorized as a socio-negative interaction that is known to have an adverse effect on bonding (e.g. [66,67]). Thus, it seems possible that the overall effect of maternal aggression was similar for both, sons and daughters. The maternal aggression resulted in weaker social bonds towards both sexes, but, in response to when the aggression was received, earlier for sons than daughters. Furthermore, we found that the context of the aggression received differed between sons and daughters. Our analysis of the interactions preceding maternal aggression revealed that aggression toward sons predominantly occurred spontaneously, i.e. maternal aggression is unlikely to be a response to immediately preceding offspring behaviour. However, as maternal responses to offspring interactions might also occur with a delay, we cannot fully exclude this possibility. In contrast, daughters frequently received maternal aggression in their second year, after handling newborns. In line with general assumptions concerning parent-offspring conflict [68], maternal aggression toward daughters may represent a shift in maternal investment from older to younger infants.

Overall, aggression was much rarer than affiliative interactions, but it has been previously suggested that aggressive interactions may have more serious consequences for bonding strength than affiliative interactions [66,69]. In red deer, in which males disperse from their natal group, maternal aggression was suggested to function as a tool to push sons to the periphery of the group [70], and maternal aggression has been found to promote offspring independence in Japanese macaques, regardless of offspring sex [71]. In addition, we have found that both adult maternal sisters and unrelated adult females in our study group–and not only mothers–direct more aggression toward male infants than to female infants in the first year of life [72].

Weaker bonds with mothers in early infancy may lead to rhesus sons searching for alternative bonding partners elsewhere. Several lines of evidence indicate that immature males in male-dispersing species indeed form bonds outside their maternal core family. In several primate species, male subadults have a higher probability than female subadults of affiliating with adult male immigrants [16,38,73]. In rhesus macaques, immature males initiate affiliation with adult males more frequently than do immature females [43]. Furthermore, immature males are more likely to affiliate with paternal kin than with non-kin in comparison to immature females and this preference starts in early infancy [74].

Weak social bonds with group members are known to stimulate natal dispersal (Social Cohesion Hypothesis [75]; as evident in e.g. [76,77]). Furthermore, studies on rhesus and Japanese macaques suggest that declining mother-son bonds are predictive of the timing of male emigration [78,79]. Timing of natal dispersal is known to be influenced by several social and environmental factors, e.g. breeding opportunities [80], dominance status [79] or population density [81]. However, despite a small sample size, our data might provide some indication that relatively higher rates of aggression and lower spatial proximity in mother-sons dyads within the first year of life tended to predict earlier natal dispersal by sons.

Mammalian mothers seem to have considerable flexibility in their behavioural strategies towards offspring. They may also bias their physical investment depending on offspring sex, by varying the sex ratio of their progeny or by transferring different proportions of their body resources to sons versus daughters [82]. While birth sex ratio adjustment is rare in mammals [83], differential transfer of maternal resources has been commonly described and is known to be biased toward sons in some species (e.g. [84,85]), but not in others (e.g. [86,87]). One commonly used measure of resource transfer in mammals is suckling behaviour (although its validity has been questioned, e.g. [88]), whereby it is assumed that offspring that suckle more receive more milk [89]. Our study found no sex differences in suckling probability between sons and daughters across development. This supports previous studies on the same species which revealed that overall milk energy received was the same for sons and daughters, although sons received richer milk and daughters more milk [50].

Notably, there are some indications from previous studies that there might be a possible link between maternal behaviour and the dispersal regime, i.e., that mothers form a stronger bond with the philopatric sex. In male philopatric chimpanzees, mothers of sons are more gregarious with other group members than the mothers of daughters [90]. As chimpanzee mothers probably influence the social development of their offspring [91], it could be argued that such higher maternal gregariousness might, in part, be a means of promoting the greater social integration of sons, who stay in their natal group throughout life [20]. In bonobos, another male-philopatric species, mothers even influence their sons’ social life in adulthood, as the behaviour of mothers affects male mating success [92] and influences dominance rank [93] of their adult sons.

In summary, our data suggest that early maternal aggression and a stronger decrease in affiliation between mothers and sons than between mothers and daughters reflect the existence of weaker mother-son bonds in rhesus macaques. In other words, the behaviour of rhesus mothers does not promote social integration of sons within their families compared to daughters, which might influence the time of male dispersal and thereby possibly consolidate female philopatry and kin bonding. In the light of a contemporary debate on cause and effect in biology [94], this study emphasizes that developmental processes potentially explain the origin of differential bonding patterns. In future studies it would be interesting to disentangle the potential effects of different bonding patterns upon dispersal, and particularly to study the ontogeny of mother-offspring bonds in male-philopatric species.

## Supporting Information

S1 File Results of GLMM for the probability of mother-offspring spatial proximity (Table A). Results of GLMM on the probability of grooming within mother-offspring dyads (Table B). Results of GLMM on the probability of aggression directed from mothers towards their offspring (Table C). Results of GLMM on the probability of nursing given by mother to their offspring (Table D). Results of GLMM on the time of natal dispersal of focal males (Table E).
(PDF)
