# Supplementary material for: Mothers Make a Difference: Mothers Develop Weaker Bonds with Immature Sons than Daughters
Source: PLoS One. 2016 May 18;11(5):e0154845. doi: 10.1371/journal.pone.0154845 (PMC4871456; doi:10.1371/journal.pone.0154845)
Supplement: S1 File — Results of GLMM for the probability of mother-offspring spatial proximity (Table A). Results of GLMM on the probability of grooming within mother-offspring dyads (Table B). Results of GLMM on the probability of aggression directed from mothers towards their offspring (Table C). Results of GLMM on the probability of nursing given by mother to their offspring (Table D). Results of GLMM on the time of natal dispersal of focal males (Table E). (PDF) [file pone.0154845.s001.pdf]

Supplementary Material

Table A Results of GLMM for the probability of mother-offspring spatial proximity

term	Estimate	SE	χ^2	df	P
(Intercept)	2.054	0.134	(1)	(1)	(1)
ac.term	0.131	0.024	27.124	1	<0.001
N of maternal kin per day (4)	-0.111	0.080	1.831	1	0.176
maternal age (4)	0.391	0.098	15.346	1	<0.001
maternal rank (4)	-0.150	0.076	3.767	1	0.052
younger infant (0=yes; 1 =no)	-0.382	0.121	9.965	1	0.002
focal age (3)(4)	-1.384	0.128	(1)	(1)	(1)
focal age^2 (3)(4)	0.387	0.092	(1)	(1)	(1)
focal sex (0=female; 1 = male)	-0.409	0.161	(1)	(1)	(1)
focal age (3)(4):focal sex (0=♀; 1 = ♂)	-0.725	0.142	27.950(2)	2(2)	<0.001(2)
focal age^2 (3)(4):focal sex (0=♀; 1 = ♂)	0.019	0.130			

Table B Results of GLMM on the probability of grooming within mother-offspring dyads

term	Estimate	SE	χ^2	df	P
(Intercept)	-0.547	0.092	(1)	(1)	(1)
ac.term	-0.016	0.024	3.818	1.000	0.051
N of maternal kin per day (4)	-0.008	0.057	0.014	1.000	0.907
maternal age (4)	0.068	0.068	0.139	1.000	0.710
maternal rank (4)	-0.069	0.053	2.325	1.000	0.127
younger infant (0=yes; 1 =no)	-0.078	0.088	0.912	1.000	0.340
focal age (3)(4)	-0.134	0.080	(1)	(1)	(1)
focal age^2 (3)(4)	-0.223	0.043	(1)	(1)	(1)
focal sex (0=female; 1 = male)	-0.321	0.116	(1)	(1)	(1)
focal age (3)(4):focal sex (0=♀; 1 = ♂)	-0.350	0.095	10.581(2)	2(2)	0.005(2)
focal age^2 (3)(4):focal sex (0=♀; 1 = ♂)	-0.073	0.062			

Table C Results of GLMM on the probability of aggression directed from mothers towards their offspring

term	Estimate	SE	χ^2	df	P
(Intercept)	-3.892	0.211	(1)	(1)	(1)
ac.term	0.247	0.050	18.61	1	<0.001
N of maternal kin per day (4)	0.086	0.104	0.74	1	0.390
maternal age (4)	-0.014	0.117	0.02	1	0.899
maternal rank (4)	-0.122	0.087	2.11	1	0.147
younger infant (0=yes; 1 =no)	0.111	0.261	0.20	1	0.657
focal age (3)(4)	0.338	0.205	(1)	(1)	(1)
focal age^2 (3)(4)	-0.385	0.140	(1)	(1)	(1)
focal sex (0=female; 1 = male)	-0.220	0.230	(1)	(1)	(1)
focal age (3)(4):focal sex (0=♀; 1 = ♂)	-0.762	0.223	12.640(2)	2(2)	0.002(2)
focal age^2 (3)(4):focal sex (0=♀; 1 = ♂)	0.072	0.196			

Table D Results of GLMM on the probability of nursing given by mother to their offspring

term	Estimate	SE	χ^2	df	P
(Intercept)	-2.389	0.219	(1)	(1)	(1)
ac.term	0.243	0.027	73.541	1	<0.001
N of maternal kin per day (4)	0.042	0.146	0.081	1	0.776
maternal age (4)	-0.025	0.174	0.020	1	0.887
maternal rank (4)	0.110	0.129	0.706	1	0.401
younger infant (0=yes; 1 =no)	-2.710	0.349	89.156	1	<0.001
focal age (3)(4)	-4.041	0.331	128.917	1	<0.001
focal age^2 (3)(4)	-0.693	0.170	14.803	1	<0.001
focal sex (0=female; 1 = male)	0.356	0.267	1.721	1	0.190

Table E Results of GLMM on the time of natal dispersal of focal males.

term	Estimate	SE	χ^2	df	P
(Intercept)	4.162	0.034	(1)	(1)	(1)
rate of maternal aggression received in the 1st year of life (4)	-0.070	0.041	2.978	1	0.084
rate of shared proximity between mother and son in the 1st year of life (4)	0.142	0.036	15.741	1	<0.001
maternal rank (4)	0.149	0.041	13.039	1	<0.001

(1): not shown because of not having a meaningful interpretation
(2): values shown for interaction comprising focal age, focal age^2 and focal sex
(3): square root transformed prior to being z-transformed
(4): predictors were z-transformed to a mean=0 and sd=1
